# Identification and Characterization of Linear Epitopes of Monoclonal Antibodies Against African Horse Sickness Virus Serotype 1 VP2 Protein

**DOI:** 10.3390/v16111780

**Published:** 2024-11-15

**Authors:** Xiaohua Ma, Yingzhi Zhang, Lei Na, Ting Qi, Weiwei Ma, Xing Guo, Xue-Feng Wang, Xiaojun Wang

**Affiliations:** 1 State Key Laboratory for Animal Disease Control and Prevention, Harbin Veterinary Research Institute of Chinese Academy of Agricultural Sciences, Harbin 150069, China; xiaohuama1299@163.com (X.M.); yingzhizhang1995@163.com (Y.Z.); qiting2013@163.com (T.Q.); mww258123@163.com (W.M.); guoxing@caas.cn (X.G.); 2 College of Animal Husbandry and Veterinary Medicine, Jiangsu Vocational College of Agriculture and Forestry, Jurong 212400, China; nl2zy@163.com; 3 Institute of Western Agriculture, The Chinese Academy of Agricultural Sciences, Changji 831100, China

**Keywords:** African horse sickness virus, monoclonal antibody, VP2

## Abstract

African horse sickness (AHS) is an acute, fatal, contagious disease of animals of the family Equidae and is caused by infection with the African horse sickness virus (AHSV). Based on the outer capsid protein VP2, AHSV is classified into nine serotypes (AHSV−1 to −9) with little or no serological cross-reactivity between them. In 2020, AHS outbreaks caused by AHSV−1 were reported in Thailand and Malaysia, marking the first occurrences of AHS in Southeast Asia. However, little is known about the antigenic profile of AHSV−1 VP2. In this study, a recombinant VP2 protein was expressed in *Escherichia coli* and used as an immunogen, and three monoclonal antibodies (mAbs), designated 7D11, 10A9, and 9E7, against AHSV−1 VP2, were generated. These three mAbs were then successfully used in IFA, WB, and ELISA for the detection of AHSV−1 VP2. Two overlapping linear epitopes, ^670^NEFDFE^675^ (E670–675) recognized by 9E7 and ^670^NEFDF^674^ (E670–674) recognized by 7D11 and 10A9, were identified through truncation of GST-fused VP2. Amino acid sequence alignment shows that the ^670^NEFDFE^675^ motif is completely conserved within AHSV−1 but is highly divergent in other AHSV serotypes. Our studies provide an important tool for basic research into AHSV−1 and for the diagnosis of AHSV−1.

## 1. Introduction

African horse sickness (AHS) is an arthropod-borne viral disease of the Equidae that is acute and fatal in susceptible horses. The disease is closely associated with respiratory and circulatory lesions and is classified as a notifiable infectious disease by the World Organization for Animal Health (WOAH). The causative agent of the disease is the African horse sickness virus (AHSV), which belongs to the genus Orbivirus (family Reoviridae and subfamily Sedoreovirinae). The severity of the disease is significantly influenced by the pathogenicity of the viral strain and the host’s susceptibility. The Equidae family, which includes horses, mules, donkeys, and zebras, has been observed to exhibit varying degrees of susceptibility to the disease. Horses have been found to be the most susceptible, with case fatality rates exceeding 90%. In contrast, mules and donkeys have been observed to demonstrate comparatively lower susceptibility [1,2,3,4]. It is noteworthy that zebras, the natural reservoirs of AHSV, do not display clinical symptoms following infection. AHS is enzootic in sub-Saharan Africa, but sporadic outbreaks have occurred in northern Africa, the Middle East and Europe, and some countries in Southern Asia (e.g., Pakistan and India) [5]. In 2020, outbreaks of AHS occurred in Southeast Asia, including Thailand and Malaysia [6,7]. As of May 2023, 69 AHS-free countries or territories are recognized by WOAH [8], but the risk of introduction of the disease into these countries is increasing due to the international movement of equines, as well as the increased migration of the midge vector associated with global warming [9,10,11].

Like bluetongue virus (BTV), which is the prototype of the genus Orbivirus, AHSV that is a spherical, non-enveloped RNA virus with an icosahedral capsid is formed by three distinct concentric protein layers [5,12]. The AHSV genome is a segmented, double-stranded RNA that contains ten RNA segments, encoding seven structural proteins (VP1-7) and six nonstructural proteins (NS1, NS2, NS3, NS3a, NS4-I, and NS4-II) [13,14,15]. The structural proteins, VP2, VP3, VP5, and VP7, form the triple-layered virion capsid. VP2 and VP5 make up the outer shell of the virion, while the outer VP7 layer and the inner VP3 layer form the core particle that encloses the viral genome. The three structural proteins, VP1 (an RNA-dependent RNA polymerase), VP4 (a capping enzyme), and VP6 (a helicase and ATPase), are viral enzymes located inside the virion and form the replication complex required for viral RNA replication and transcription. These three structural proteins are encapsulated with viral dsRNA genomic segments by VP3 to form the subcore particle. The nonstructural proteins are involved in the replication, morphogenesis, and release of AHSV [5,16].

The AHSV VP2 protein is approximately 110 kDa in size and is encoded by genome segment 2 [17]. Few extensive studies have been conducted on the molecular biology of AHSV VP2, although studies of BTV have confirmed that VP2 is responsible for cell attachment and entry of the Orbivirus into the host cell [18,19]. AHSV VP2 is a major protective antigen that contains most of the known virus-neutralizing antibody epitopes [20]. Consequently, this protein is a major candidate for the development of an AHSV vaccine [21,22]. However, VP2 is a highly variable antigen and serves as the primary determinant of AHSV serotypes. The virus is classified into nine serotypes (AHSV−1 to −9) based on VP2 antigenicity, and the serological cross-reactivity induced by the nine AHSV serotypes is generally minimal, although some cross-reaction has been observed between serotypes 1 and 2, 3 and 7, 5 and 8, and 6 and 9 [23].

AHSV VP2 has been extensively studied as an important immunogen [21,24,25,26]. However, only a few studies have reported on its antigenic epitopes, and these have only been reported for AHSV−3 and −4 [27,28,29,30,31]. Monoclonal antibodies (mAbs) against viral proteins with high specificity for the antigen are indispensable tools in diagnostics and research. To our knowledge, no mAbs against ASHV−1 VP2 have been reported to date. In this study, we expressed and purified recombinant AHSV−1 VP2 protein and then generated and characterized three mAbs against VP2. Two linear VP2 B−cell epitopes were subsequently identified with Western blot (WB) analysis using these mAbs. These epitopes were found to be conserved in AHSV-1 but not in other AHSV serotypes. Our results provide the basis for the specific detection of AHSV−1 and contribute to basic research into and knowledge of AHSV−1.

## 2. Materials and Methods

### 2.1. Cells, Virus and Vectors

Primary chicken embryo fibroblast (CEF) cells were cultured at 37 °C in a humidified incubator with 5% CO_2_ using Dulbecco’s Modified Eagle’s Medium (DMEM) (Thermo Fisher Scientific, Waltham, MA, USA) supplemented with 10% heat-inactivated fetal bovine serum (FBS). The SP2/0 myeloma cell line was cultured following previously published protocols [32].

An attenuated fowlpox virus (FPV) strain (FPV-017) was maintained in our laboratory [33]. Recombinant FPV expressing AHSV-1 VP2 (rFPV-VP2) was generated based on FPV-017 (see Section 2.7 for specific preparation details). Both viruses were propagated in CEF cells in DMEM supplemented with 2% FBS.

The prokaryotic expression vectors pGEX-6P-1 and pET28a were maintained in our laboratory. An FPV transfer vector, pSY-VP2-LacZ1, was constructed based on the plasmid pSY-HA-LacZ1, previously generated in our laboratory [33]. The transfer vector contained the full-length coding sequence of AHSV-1 VP2 (GenBank accession number KT186898) under the early-late LP2EP2 promoter of FPV, as well as a LacZ gene fragment under the P11 late promoter of the vaccinia virus. Furthermore, the pSY-HA-LacZ1 contains a hemagglutinin (HA) gene from the equine influenza virus under the early-late LP2EP2 promoter of FPV and a LacZ gene fragment under the P11 late promoter of the vaccinia virus. In this study, the HA gene in pSY-HA-LacZ1 was replaced with the synthesized coding sequence of VP2 by overlap extension PCR to generate the pSY-VP2-LacZ1 plasmid.

### 2.2. Expression and Purification of Recombinant AHSV-1 VP2

The plasmid pET28a-VP2^336–825^ was generated using the pET28a vector. The partial coding sequence of AHSV-1 VP2 was inserted into the pET28a vector. This partial coding sequence corresponds to aa 336-825 of the VP2 protein (GenBank accession number ALL54664.1) and was synthesized by the Sangon Biotech Company (Shanghai, China) and optimized based on *E. coli* preferred codons. The plasmid was identified using digestion with the restriction enzymes NdeI (Thermo Fisher Scientific, Waltham, MA, FD0583, USA) and XhoI (Thermo Fisher Scientific, Waltham, MA, FD0694, USA) and was further confirmed using DNA sequencing (Comet Bioscience Co., Ltd., Jilin, China).

To produce the VP2 fusion proteins, pET28a-VP2^336–825^ was expressed in *E. coli* BL21 (DE3) competent cells. Isopropyl-β-D-1-thiogalactopyranoside (IPTG) was added at 0.1 mM or 0.5 mM and was induced overnight at different temperatures (16 °C, 25 °C, 37 °C) to express the proteins. Then the expressed proteins in the precipitation and supernatant were analyzed with sodium dodecyl sulfate-polyacrylamide gel electrophoresis (SDS-PAGE) following ultrasound analysis. Bacterial cells were then centrifuged at 5000× *g* for 10 min, washed sequentially with wash buffer 1 (2 M urea) and wash buffer 2 (50 mmol/L Tris-HCl, 10 mmol/L EDTA, 100 mmol/L NaCl, 0.5% TritonX-100), and were then resuspended in PBS to prepare SDS-PAGE protein samples. After electrophoresis, the gel was stained with 0.25 mM KCl. The silver-white stained bands containing the target proteins were carefully excised and placed in a dialysis bag. Then, 5 mL of protein electrophoresis solution (1X Tris-MOPS-SDS running buffer) was added, and the dialysis bag was tightly sealed and placed in 1X Tris-acetate-EDTA (TAE) solution at 180 V for 1 h to separate the proteins from the gel. The target protein (His-VP2^336–825^) was released in the protein electrophoresis solution and then concentrated using dialysis with PBS. Here, we designate this method of protein purification as the gel-excising purification method. The purified proteins were then analyzed with SDS-PAGE and WB assays.

To identify the epitope of the prepared mAbs, the sequence of VP2^336–825^ was divided into three partially overlapping fragments. A series of truncated fragments was then constructed, and these were cloned separately into the pGEX-6P-1 vector. The reactivity of the mAbs against these different fragments was then assessed using WB.

### 2.3. Preparation of mAbs

Six-week-old female BALB/c mice were inoculated in the leg muscles with the purified recombinant His-VP2^336–825^ protein mixed with complete/incomplete Freund’s adjuvant at 0.1 mg per mouse at three-week intervals. After assessment with indirect ELISA (iELISA), the mice with the highest antibody levels were selected. To sustain the immune response, the protein was injected subcutaneously 72 h prior to the fusion. Splenocytes from the immunized mice were fused with SP2/0 myeloma cells in the presence of PEG solution (Sigma-Aldrich, St. Louis, MO, USA). The fused cells were then cultured in 96-well plates using HAT (hypoxanthine, aminopterin, thymidine) selection medium (Sigma-Aldrich, St. Louis, MO, USA). After seven days, positive hybridoma cells were identified using iELISA. Hybridoma supernatants were collected, and the antibody responses were analyzed with iELISA coated with recombinant His-VP2^336–825^ protein, and 6×His-tagged small ruminant lentivirus capsid protein (p28) was used as a specificity control [32]. After three rounds of subcloning, single cells exhibiting higher OD_450_ nm values were expanded and cultured. The hybridoma cells producing stable mAbs were identified using WB. To determine the subtype of the mAbs, a commercial antibody subtype identification kit (Southern Biotech, Birmingham, AL, USA) was employed. Freund’s incomplete adjuvant and selected hybridoma cells were injected three days apart to produce and collect ascites. The ascites were then purified using an antibody purification kit (GE Healthcare, Chicago, IL, USA). The specificity of the prepared mAbs was subsequently analyzed with WB and IFA.

### 2.4. iELISA

The His-VP2^336–825^ antigen was diluted in CBS (pH 9.6) to a concentration of 200 ng/well and was subsequently added to 96-well polystyrene plates. The plates were incubated overnight at 4 °C to facilitate antigen adsorption. Following this incubation, the plates were washed three times with PBST (0.05% Tween in PBS, *v*/*v*) and then blocked with 200 µL of 5% skimmed milk in PBS for 2 h at 37 °C to prevent non-specific binding. Samples, including hybridoma supernatants or purified mAbs, were added to the wells and were incubated for 2 h at 37 °C. After washing the plates three additional times with PBST to remove unbound samples, 100 µL of horseradish peroxidase (HRP)-conjugated goat anti-mouse IgG (KPL, Gaithersburg, MD, USA), diluted 1:5000 in PBS, was added to each well, and samples were incubated for 40 min at 37 °C. After a further wash with PBST, 100 µL of chromogenic substrate solution (TMB) (Beyotime Biotechnology Co., Ltd., Shanghai, China) was added to each well, and the reaction was allowed to develop for 10 min in the dark while color change was monitored. To halt the reaction, 50 µL of stop solution (2 M H_2_SO_4_) was added to each well. Finally, the absorbance was measured at 450 nm using an automated ELISA plate reader (Bio Tek, Winooski, VT, USA).

### 2.5. Western Blot

To evaluate the reactivity of the prepared mAbs with recombinant VP2 protein or the minimal epitope required for recognizing VP2, the corresponding protein samples were analyzed with WB. The experiments were conducted according to previously reported methods [32]. The anti-VP2 mAbs (hybridoma supernatant) served as the primary antibody for this project, and anti-GST and anti-His antibodies were utilized as positive controls, anti-mouse IgG-DyLight 800 (1:5000 dilution) was employed as the secondary antibody, and membranes were visualized using the LI-COR Odyssey Imagining System (LI-COR, Lincoln, NE, USA).

To assess the reactivity of the prepared mAbs with eukaryotic full-length VP2 proteins, CEF cell layers were infected with rFPV-VP2 and FPV017 at a multiplicity of infection (MOI) of 0.1, and the cells were harvested and lysed 96 h post-infection. Cell lysates were subjected to electrophoresis on a 12% SDS-PAGE gel, followed by WB as described above.

### 2.6. Immunofluorescence Assay (IFA)

To evaluate antibody reactivity, CEF cell layers were infected with rFPV-VP2 and FPV017 at a multiplicity of infection (MOI) of 0.1. Following a 96 h culture period, the procedure adhered to previously established methods [32], with the exception that the primary antibodies used were the prepared mAbs and the secondary antibody was FITC-conjugated goat anti-mouse IgG (whole molecule) (Sigma-Aldrich, St. Louis, MO, USA). Fluorescent signals were observed using the EVOS M5000 inverted fluorescence microscope (Life, Bothell, WA, USA), enabling the analysis of antibody responses.

### 2.7. Generation of rFPV-VP2

The rFPV-VP2 was generated as described previously [33,34]. Briefly, CEF cells were infected with S-FPV-017 and then transfected with the pSY681-VP2-LacZ plasmid. The cells were incubated at 37 °C for 3–5 days until the cytopathic effect appeared. Then the cells were repeatedly frozen and thawed three times and used as seed virus stock for screening. A monolayer of CEF cells was infected with the different concentrations of the seed virus stock and then subjected to plaque purification. After eight rounds of consecutive blue plaque purification, rFPV-VP2 was obtained and expanded in CEF cells. The insertion of VP2 into rFPV-VP2 was also confirmed using PCR and WB.

### 2.8. Alanine-Scanning Mutagenesis

To characterize the key amino acids of epitope E670–675 that bind to the prepared mAbs, each amino acid within the ^670^NEFDFE^675^ motif was separately mutated to alanine using PCR-based point mutation with pGEX6p-E670–675 as a template. All mutants with a single alanine substitution were expressed in *E. coli*. Bacterial lysates were collected using sonication and analyzed for reactivity with the prepared mAbs using WB.

### 2.9. Conservation Analysis of the Identified Epitopes

To analyze the conservation of the epitope E670–675, the VP2 protein sequences of all nine serotypes of AHSV were downloaded from Genbank and were aligned using the DNASTAR v7.1 software (DNASTAR, Madison, WI, USA). The nucleotide sequences of the nine serotypes of AHSV corresponding to the epitope E670–675 were synthesized and cloned into the pGEX-6P-1 vector and expressed in *E. coli*. Bacterial lysates were collected after sonication and analyzed using WB.

### 2.10. Biological Information Analysis

The DNASTAR v7.1 software (DNASTAR, Madison, WI, USA) was used to analyze the secondary structural domain of the AHSV−1 VP2. The VP2 structure was predicted using the high-precision protein 3D structure prediction system Alphafold3 (https://alphafoldserver.com/) (accessed on 4 September 2024). The spatial characteristics of the identified epitope in the VP2 protein were analyzed by mapping the epitope positions onto the model of the VP2 protein using the PyMOL software v3.1 (Schrodinger, New York, NY, USA).

## 3. Results

### 3.1. Expression and Purification of the Recombinant His-VP2^336–825^ Protein

**Figure 1:**
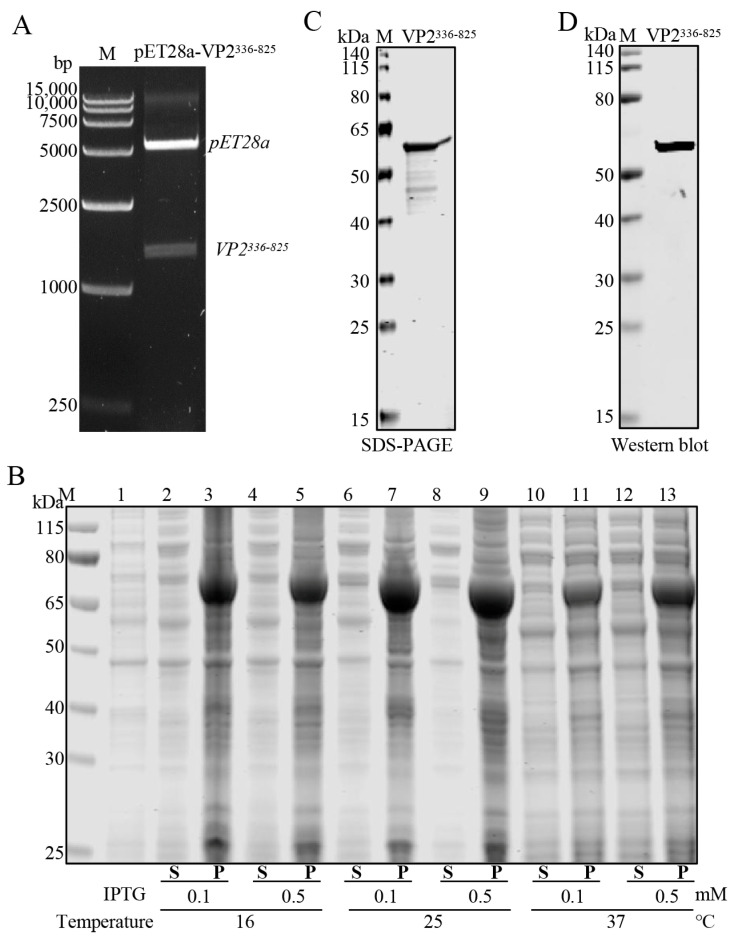
Expression and purification of recombinant His-tagged AHSV−1 VP2^336–825^. (**A**) Identification of recombinant plasmid pET28a-VP2^336–825^ using Nde I/Xho I digestion. Lane M: DNA molecular weight marker. (**B**) SDS-PAGE analysis showing the expression of recombinant His-VP2^336–825^ protein. Lane M, protein marker; lane 1, lysates from *E. coli* BL21 (DE3) transformed with the empty vector pET28a; lanes 2–13, supernatant (S) and precipitation (P) from *E. coli* BL21 (DE3) cells transformed with pET28a-VP2^336–825^, induced under different conditions as indicated at the bottom of the figure. (**C**) The purified His-VP2^336–825^ protein was analyzed using SDS-PAGE and Coomassie Brilliant Blue staining. (**D**) The purified His-VP^2336–825^ protein was subjected to WB analysis using an anti-His antibody.

A 1470 bp nucleotide sequence encoding the amino acid sequence corresponding to region 336-825 aa of the full-length AHSV−1 VP2 protein was synthesized and cloned into pET-28a. Recombinant-positive plasmids were identified using restriction enzyme digestion (Figure 1A) and sequencing. The recombinant His-VP2^336–825^ protein was expressed in *E. coli* BL21 (DE3) cells. SDS-PAGE demonstrated that the His-VP2^336–825^ protein was expressed with a predicted molecular mass of approximately 65 kDa and was mainly present in a predominantly insoluble form in the precipitation of cell lysate after sonication (Figure 1B). High-purity recombinant His-VP2^336–825^ protein was subsequently obtained (Figure 1C), and WB analysis was used to demonstrate that the purified His-VP2^336–825^ protein reacted specifically with the anti-His antibody (Figure 1D).

### 3.2. Preparation and Characterization of mAbs Against AHSV-1 VP2

**Figure 2:**
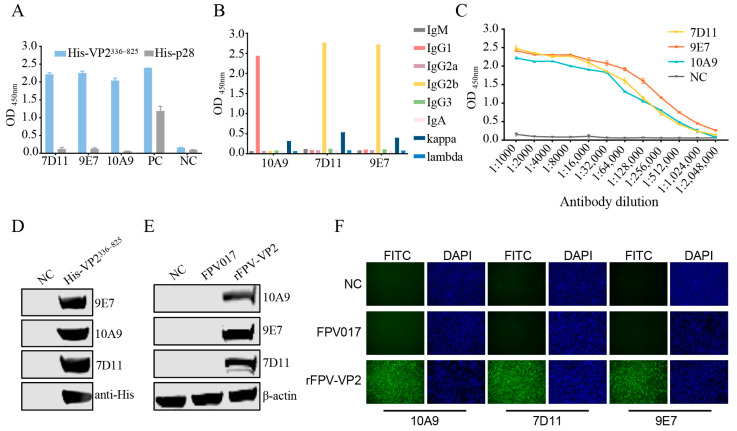
Screening and characterization of the mAbs. (**A**) Reactivity of the prepared mAbs with recombinant VP2^336–825^ protein was assessed using iELISA, with His-p28 protein as a specificity control. An anti-His antibody was used as a positive control (PC), and a serum from unimmunized mice was used as a negative control (NC). Each sample was tested in triplicate. (**B**) Isotype identification of prepared mAbs. (**C**) Antibody titers of the prepared mAbs were assessed using iELISA. A serum from an unimmunized mouse was used as an NC. Each sample was tested in triplicate. (**D**) Reactivity of the prepared mAbs with prokaryotic recombinant VP2^336–825^ protein was assessed with WB. The empty vector pET28a was used as an NC. (**E**,**F**) Reactivity of the prepared mAbs with eukaryotic VP2 protein was assessed with WB (**E**) and IFA (**F**). Uninfected cells served as the NC.

Three hybridoma cell lines, designated 7D11, 10A9, and 9E7, secreting antibodies against the AHSV−1 VP2 were obtained (Figure 2A). Antibody isotype assays determined that mAb 10A9 belonged to the IgG1κ type and mAbs 7D11 and 9E7 belonged to the IgG2bκ type (Figure 2B). Analysis with iELISA demonstrated that the titers of mAb 10A9 at a concentration of 2 μg/mL were above 1,024,000 and those of mAbs 9E7 and 7D11 were above 2,048,000 (Figure 2C). Subsequent WB assays showed that all three mAbs specifically recognized prokaryotically expressed His-VP2^336–825^ (Figure 2D). In addition, we used WB (Figure 2E) and IFA (Figure 2F) to confirm that all three mAbs specifically recognized the full-length VP2 protein expressed in rFPV-VP2-infected CEF cells. These results suggest that the three mAbs recognize the linear epitope of AHSV−1 VP2.

### 3.3. Identification of the Epitopes Recognized by the Three mAbs

**Figure 3:**
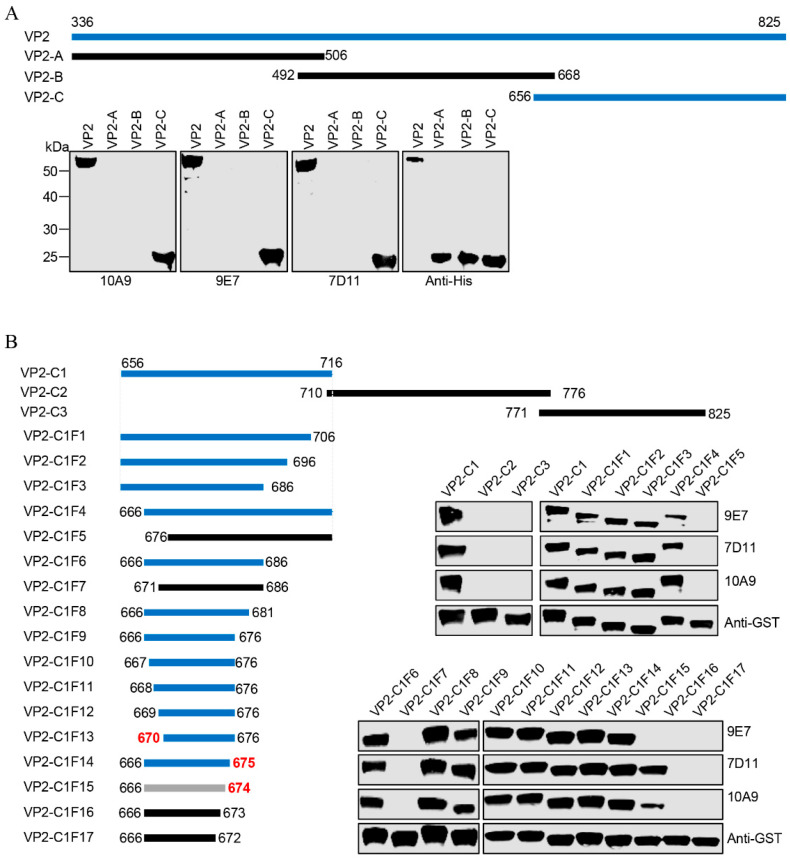
Mapping of the linear B-cell epitopes of AHSV-1 VP2 with the prepared mAbs. (**A**) Reactivity of the segmented expressed VP2 protein with the indicated mAbs was determined using WB. An anti-6xHis antibody was used to determine the loading dose of each sample. The blue lines represent VP2 fragments that react with the mAbs, the black lines represent VP2 fragments that do not react with either mAb. The numbers represent the positions of the amino acids in the full-length VP2 (GenBank accession number ALL54664). (**B**) Reactivity of various GST-tagged VP2 truncated mutants with the indicated mAbs was tested with WB. An anti-GST antibody was used to determine the loading dose of each sample. The gray lines represent VP2 fragments that react only with specific mAbs, namely 7D11 and 10A9. The red numbers indicate the N-terminal or C-terminal location of the minimal fragment recognized by the mAbs.

**Figure 4:**
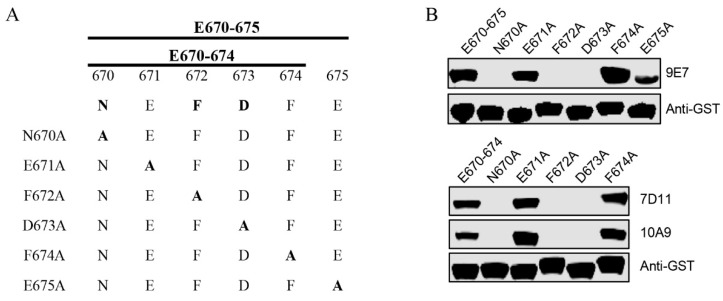
Alanine scanning to determine the key amino acids of E670–675 and E670–674. (**A**) This schematic illustrates the mutation strategy. (**B**) Six mutated GST-tagged E670–675 proteins (upper) and five mutated GST-tagged E670–674 proteins (lower) were expressed and tested for reactivity with the indicated mAbs using WB. GST-E670–675 and GST-E670–674 were used as positive controls. An anti-GST antibody was used to determine the loading dose of each sample.

To identify the epitopes recognized by the mAbs 7D11, 10A9, and 9E7, the VP2^336–825^ fragment was subdivided into three overlapping fragments (VP2-A, VP2-B, and VP2-C). WB assays showed that all three mAbs recognized only the VP2-C fragment (Figure 3A). Subsequently, a series of truncated VP2 proteins fused with GST tags were designed. WB assays demonstrated that the minimal linear epitopes recognized by these mAbs were located at ^670^NEFDF^674^ (recognized by 7D11 and 10A9, designated E670–674) and ^670^NEFDFE^675^ (recognized by 9E7, designated E670–675) of AHSV-1VP2 (Figure 3B). We also confirmed that the GST-^670^NEFDF^674^ fusion protein was recognized by 7D11 and 10A9, and the GST-^670^NEFDFE^675^ fusion protein was recognized by 9E7 (Figure 4). These results demonstrate that two overlapping antigenic epitopes of AHSV-1 VP2 were identified.

### 3.4. Identification of Critical Amino Acids Within the E670–675 and E670–674 for Recognition by the mAbs

To identify the critical residues of the epitopes recognized by the three mAbs, we performed an alanine scan based on the epitopes E670–675 and E670–674 (Figure 4A). WB analysis revealed that substitution of Asn670, Phe672, or Asp673 with alanine completely abrogated binding of these two epitopes to their corresponding mAbs, whereas substitutions at other positions did not (Figure 4B). This suggests that the residues Asn670, Phe672, and Asp673 are critical for the interaction of epitopes E670–675 and E670–674 with these mAbs.

### 3.5. Conservative Analysis of the Epitopes

**Figure 5:**
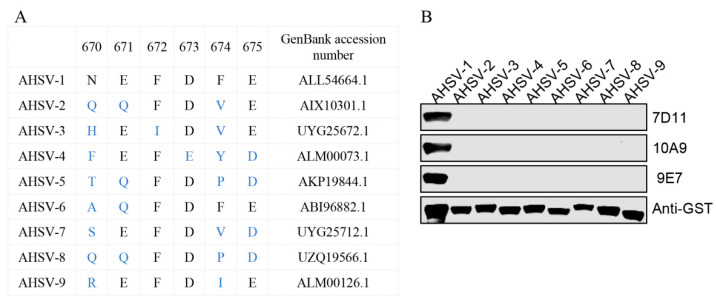
Conservative analysis of the epitopes. (**A**) This schematic illustrates the amino acid sequences of E670–675 (corresponding to AHSV−1 VP2) and the corresponding region in VP2 of AHSV−2 to 9. The black letters represent sites identical to the reference sequence (AHSV−1), while the blue letters indicate sites inconsistent with the reference sequence (AHSV−1). (**B**) The reactivity of a series of GST-fusion NEFDFE proteins containing the mutation site indicated in panel A was analyzed with 7D11, 10A9 and 9E7 using WB. An anti-GST antibody was used to determine the loading dose of each sample.

To assess the conservation of E670–675 and E670–674 among AHSV VP2, a total of 165 VP2 amino acid sequences of serotype-defining AHSV (including AHSV−1 to 9) were downloaded from Genbank and aligned. We found that the ^670^NEFDFE^675^ motif was completely conserved within AHSV-1, but there was high divergence in AHSV 2 to 9 (Supplementary Figure S1). GST fusion proteins containing the ^670^NEFDFE^675^ motif or mutant motifs corresponding to the VP2 of AHSV serotypes 2 to 9 were prepared (Figure 5A). WB analysis showed that the three mAbs recognized only the GST-NEFDFE fusion protein (corresponding to AHSV-1), but not other mutants (corresponding to AHSV−2 to −9) (Figure 5B), indicating that these mAbs can specifically recognize only AHSV−1 VP2, but not VP2 from other serotypes of AHSV. Subsequently, we performed a protein-protein blast using the NEFDFE motif, which revealed that the motif is not only present in AHSV−1 but also in certain microorganisms or species. However, these microorganisms or species are not associated with equine pathogens or equine-derived proteins (Supplementary Figure S2). The results suggest that these mAbs have the potential to be used for the specific detection of AHSV−1 in equine animals.

### 3.6. Prediction of the Spatial Structures and Biological Characteristics of VP2 Epitope E670–675

**Figure 6:**
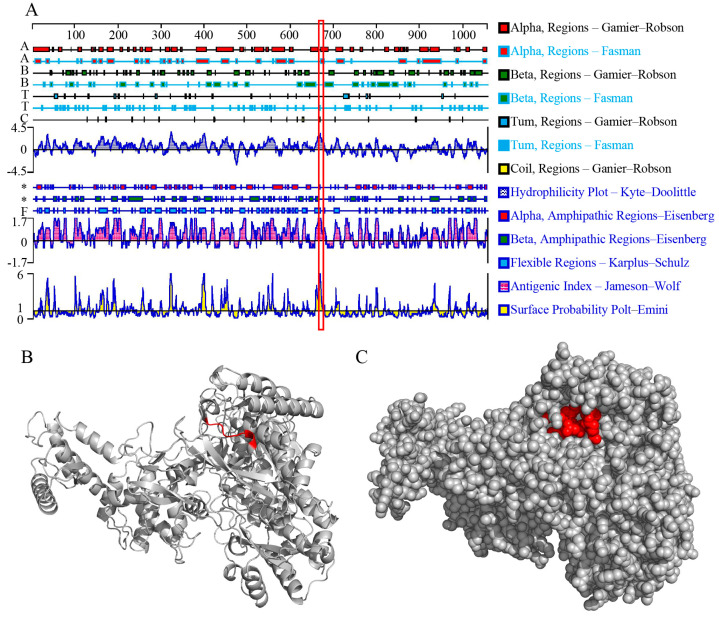
Structural analysis of the AHSV−1 VP2 protein. (**A**) The secondary structure and biological properties of antigenic epitopes of AHSV−1 VP2 proteins. (**B**,**C**) The spatial position of epitope E670−675 on the predicted 3D structure (cartoon model, B; sphere model, C) of AHSV−1 VP2.

The secondary structural domains and three-dimensional structure of the full-length AHSV−1 VP2 protein were predicted and modeled using online resources and biological software. The epitope E670–675 (^670^NEFDFE^675^) exhibits significant hydrophilicity, a high antigen index, and forms an α-helix (Figure 6A). The three-dimensional model indicates that the epitope is situated on the surface of the VP2 protein (Figure 6B,C), further indicating that the epitope is linear and immunogenic.

## 4. Discussion

VP2 of AHSV is the serotype-specific protein of AHSV and is also a major structural protein [21,23,24,25]. mAbs are extremely valuable tools for both basic research and the diagnosis of pathogenic microorganisms. To date, only a few mAbs against AHSV VP2 have been reported, mainly against the VP2 of AHSV−3 and −4 [35,36], while no mAb against the VP2 of AHSV−1 has been reported. In the present study, we generated three mAbs against the AHSV−1 VP2, using the recombinant VP2^336–825^ protein expressed in *E. coli* as an immunogen, and then characterized the epitope sequences recognized by these three mAbs. To obtain mAbs against AHSV−1 VP2, we attempted to express the full-length AHSV−1 VP2 in a prokaryotic system but were unsuccessful. A similar observation was also reported in the study by Wang et al. [37]. Subsequently, a fragment located in amino acids 336–825 of AHSV−1 VP2 was expressed and purified in *E. coli* and was used as an immunogen to immunize mice, resulting in three mAbs being obtained. A previous study showed that the major antigenic domain of the AHSV−4 VP2 is located in a central region (amino acids 200 to 413) and that neither the N-terminal region nor the half C-terminal region are immunogenic [28]. Several recent studies have demonstrated the presence of numerous B-cell epitopes on AHSV−4 VP2. Mathebula et al. reported that there are three antigenic regions on AHSV−4 VP2, the first two near the N-terminus (amino acids 20 to 63, amino acids 123 to 150) and one at the C-terminus (amino acids 1036 to 1054) [31]. Two B-cell epitopes located at amino acids 321–352 and 928–959 of AHSV−4 VP2 were also identified by Faber et al. [30]. Interestingly, a recent study has shown that the N-terminus (amino acids 1 to 502) and the C-terminus (amino acids 503 to 1065) of AHSV−1 VP2 induced specific humoral and cellular immune responses in mice [37]. Therefore, it is possible that the recombinant VP2^336–825^ produced in this study could be used as a detection antigen in the serological diagnosis of AHSV−1 infection.

Previously, a number of studies have reported that the full-length AHSV VP2 protein can be expressed in eukaryotic systems, including the baculovirus expression system [25,38] and poxvirus vector [39,40]. Due to the lack of naive AHSV−1 samples in our laboratory, an rFPV-VP2 was used here to confirm that the three mAbs specifically recognize the full-length AHSV−1 VP2 protein. We also demonstrated that these three mAbs can be used in iELISA, IFA, and WB assays to detect AHSV−1 VP2. In addition, we confirmed that the epitope sequences recognized by these mAbs are highly conserved among AHSV−1. These results suggest that these three mAbs have the potential to serve as important tools for basic research and diagnosis of AHSV−1.

Although several previous studies have identified several B-cell epitopes of AHSV−4 VP2 [27,30,31], the B-cell epitope of AHSV−1 VP2 has not yet been reported. In this study, we found that all three mAbs recognize AHSV−1 VP2 expressed in both prokaryotic and eukaryotic systems and are suitable for use in both WB and IFA analyses, indicating that they recognize linear B-cell epitopes. To accurately map the linear epitopes, a series of GST-fused VP2 segments expressed in *E. coli* were generated. WB assays showed that these three mAbs (7D11, 10A9, and 9E7) recognize two overlapping epitopes, i.e., 7D11 and 10A9 recognize E670–674 (^670^NEFDF^674^), and 9E7 recognizes E670–675 (^670^NEFDFE^675^). However, 7D11 and 10A9 belong to the IgG2bκ and IgG1κ isotypes, respectively, although they recognize the same epitope (E670–674). This suggests that the motif ^670^NEFDFE^674^ may be the major immunorecognition region of AHSV−1 VP2. Unfortunately, we were unable to test the reactivity of these epitopes with AHSV−1-positive horse serum due to the lack of this serum in our laboratory. Several studies have shown that the neutralizing epitope of AHSV is located in the VP2 [27,28,41]. Therefore, further studies are needed to determine whether these three mAbs have neutralizing activity. The VP2 is the most variable AHSV protein, and the homology of VP2 protein sequences between all nine AHSV serotypes varied between 47.6% and 71.4% [38]. We found that these two epitopes are highly divergent between different serotypes of AHSV VP2. However, these epitopes are highly conserved within the AHSV−1 VP2. In addition, the 3D spatial structure shows that the epitopes are exposed on the surface of the VP2. These results suggest that these epitopes could be developed as targets for the serological detection of AHSV−1 and that the mAbs have the potential to serve as important tools for the differential diagnosis of AHSV−1.

In summary, we generated for the first time mAbs against AHSV−1 VP2 and characterized the epitope sequences they recognize. These three mAbs were successfully used in IFA, WB, and ELISA for the detection of AHSV−1 VP2. Our studies provide an important tool for basic research into AHSV−1 and the diagnosis of AHSV−1.

## Data Availability

All required data are available in the manuscript. Any additional data can be provided upon request.

## Supplementary Materials

The following supporting information can be downloaded at: https://www.mdpi.com/article/10.3390/v16111780/s1, Figure S1: Analysis of the conservation of the ^670^NEFDFE675 epitope in nine serotypes of AHSV; Table S1: List of species containing the NEFDFE motif from NCBI protein databases using protein-protein blast.
