# Supplementary material for: Identification and Characterization of Linear Epitopes of Monoclonal Antibodies Against African Horse Sickness Virus Serotype 1 VP2 Protein
Source: Viruses. 2024 Nov 15;16(11):1780. doi: 10.3390/v16111780 (PMC11599129; doi:10.3390/v16111780)
Supplement: Supplementary file 1 [file viruses-16-01780-s001.zip › Figure. S1.pptx]

## Slide 1
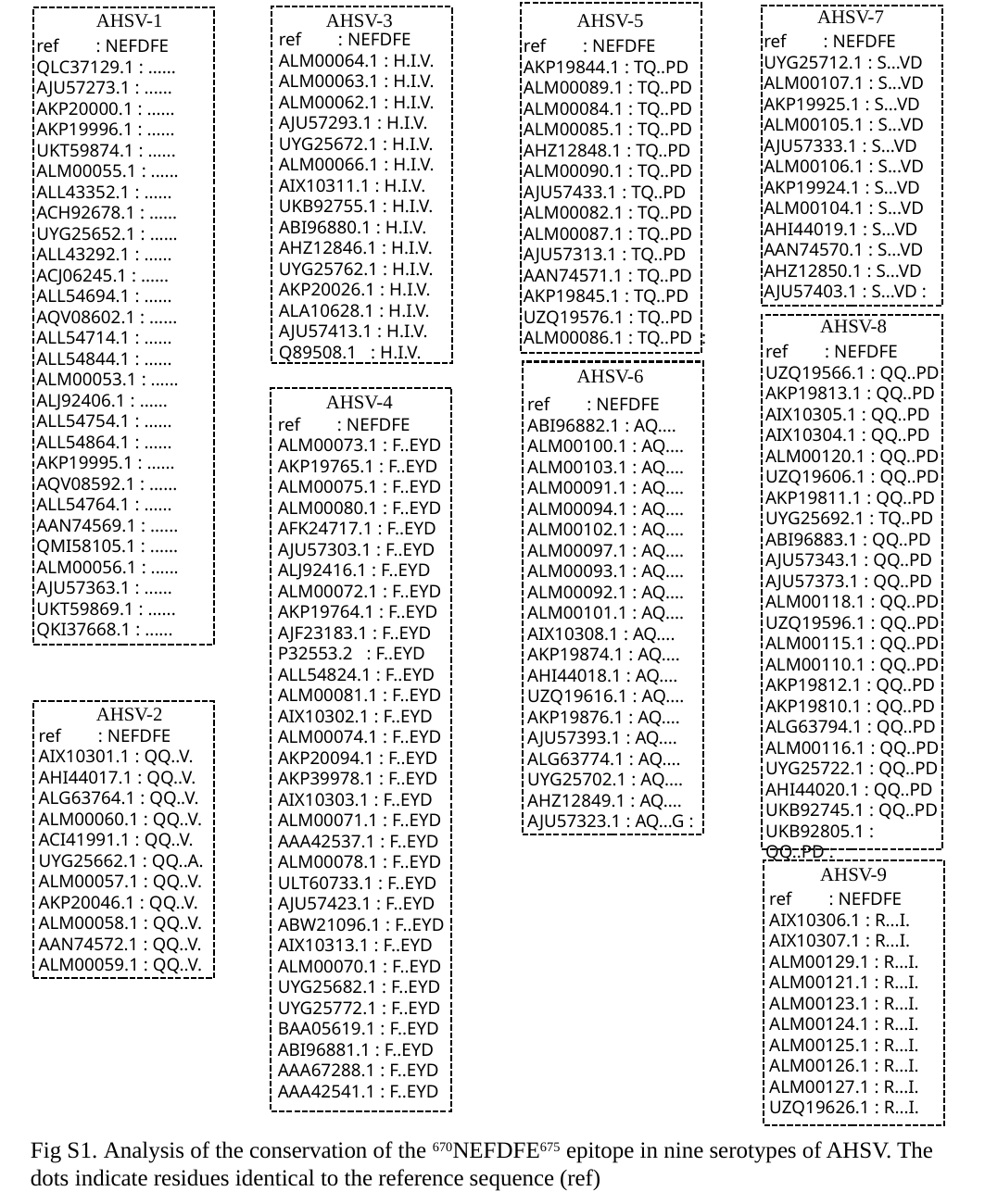

ref : NEFDFE
ALM00064.1 : H.I.V.
ALM00063.1 : H.I.V.
ALM00062.1 : H.I.V.
AJU57293.1 : H.I.V.
UYG25672.1 : H.I.V.
ALM00066.1 : H.I.V.
AIX10311.1 : H.I.V.
UKB92755.1 : H.I.V.
ABI96880.1 : H.I.V.
AHZ12846.1 : H.I.V.
UYG25762.1 : H.I.V.
AKP20026.1 : H.I.V.
ALA10628.1 : H.I.V.
AJU57413.1 : H.I.V.
Q89508.1 : H.I.V.
ref : NEFDFE
UYG25712.1 : S...VD
ALM00107.1 : S...VD
AKP19925.1 : S...VD
ALM00105.1 : S...VD
AJU57333.1 : S...VD
ALM00106.1 : S...VD
AKP19924.1 : S...VD
ALM00104.1 : S...VD
AHI44019.1 : S...VD
AAN74570.1 : S...VD
AHZ12850.1 : S...VD
AJU57403.1 : S...VD :
AHSV-7
ref : NEFDFE
QLC37129.1 : ......
AJU57273.1 : ......
AKP20000.1 : ......
AKP19996.1 : ......
UKT59874.1 : ......
ALM00055.1 : ......
ALL43352.1 : ......
ACH92678.1 : ......
UYG25652.1 : ......
ALL43292.1 : ......
ACJ06245.1 : ......
ALL54694.1 : ......
AQV08602.1 : ......
ALL54714.1 : ......
ALL54844.1 : ......
ALM00053.1 : ......
ALJ92406.1 : ......
ALL54754.1 : ......
ALL54864.1 : ......
AKP19995.1 : ......
AQV08592.1 : ......
ALL54764.1 : ......
AAN74569.1 : ......
QMI58105.1 : ......
ALM00056.1 : ......
AJU57363.1 : ......
UKT59869.1 : ......
QKI37668.1 : ......
ref : NEFDFE
AKP19844.1 : TQ..PD
ALM00089.1 : TQ..PD
ALM00084.1 : TQ..PD
ALM00085.1 : TQ..PD
AHZ12848.1 : TQ..PD
ALM00090.1 : TQ..PD
AJU57433.1 : TQ..PD
ALM00082.1 : TQ..PD
ALM00087.1 : TQ..PD
AJU57313.1 : TQ..PD
AAN74571.1 : TQ..PD
AKP19845.1 : TQ..PD
UZQ19576.1 : TQ..PD
ALM00086.1 : TQ..PD :
AHSV-1
AHSV-3
AHSV-5
ref : NEFDFE
UZQ19566.1 : QQ..PD
AKP19813.1 : QQ..PD
AIX10305.1 : QQ..PD
AIX10304.1 : QQ..PD
ALM00120.1 : QQ..PD
UZQ19606.1 : QQ..PD
AKP19811.1 : QQ..PD
UYG25692.1 : TQ..PD
ABI96883.1 : QQ..PD
AJU57343.1 : QQ..PD
AJU57373.1 : QQ..PD
ALM00118.1 : QQ..PD
UZQ19596.1 : QQ..PD
ALM00115.1 : QQ..PD
ALM00110.1 : QQ..PD
AKP19812.1 : QQ..PD
AKP19810.1 : QQ..PD
ALG63794.1 : QQ..PD
ALM00116.1 : QQ..PD
UYG25722.1 : QQ..PD
AHI44020.1 : QQ..PD
UKB92745.1 : QQ..PD
UKB92805.1 : QQ..PD :
AHSV-8
ref : NEFDFE
ALM00073.1 : F..EYD
AKP19765.1 : F..EYD
ALM00075.1 : F..EYD
ALM00080.1 : F..EYD
AFK24717.1 : F..EYD
AJU57303.1 : F..EYD
ALJ92416.1 : F..EYD
ALM00072.1 : F..EYD
AKP19764.1 : F..EYD
AJF23183.1 : F..EYD
P32553.2 : F..EYD
ALL54824.1 : F..EYD
ALM00081.1 : F..EYD
AIX10302.1 : F..EYD
ALM00074.1 : F..EYD
AKP20094.1 : F..EYD
AKP39978.1 : F..EYD AIX10303.1 : F..EYD
ALM00071.1 : F..EYD
AAA42537.1 : F..EYD
ALM00078.1 : F..EYD
ULT60733.1 : F..EYD
AJU57423.1 : F..EYD
ABW21096.1 : F..EYD
AIX10313.1 : F..EYD
ALM00070.1 : F..EYD
UYG25682.1 : F..EYD
UYG25772.1 : F..EYD
BAA05619.1 : F..EYD
ABI96881.1 : F..EYD
AAA67288.1 : F..EYD
AAA42541.1 : F..EYD
ref : NEFDFE
ABI96882.1 : AQ....
ALM00100.1 : AQ....
ALM00103.1 : AQ....
ALM00091.1 : AQ....
ALM00094.1 : AQ....
ALM00102.1 : AQ....
ALM00097.1 : AQ....
ALM00093.1 : AQ....
ALM00092.1 : AQ....
ALM00101.1 : AQ....
AIX10308.1 : AQ....
AKP19874.1 : AQ....
AHI44018.1 : AQ....
UZQ19616.1 : AQ....
AKP19876.1 : AQ....
AJU57393.1 : AQ....
ALG63774.1 : AQ....
UYG25702.1 : AQ....
AHZ12849.1 : AQ....
AJU57323.1 : AQ...G :
AHSV-6
AHSV-4
ref : NEFDFE
AIX10301.1 : QQ..V.
AHI44017.1 : QQ..V.
ALG63764.1 : QQ..V.
ALM00060.1 : QQ..V.
ACI41991.1 : QQ..V.
UYG25662.1 : QQ..A.
ALM00057.1 : QQ..V.
AKP20046.1 : QQ..V.
ALM00058.1 : QQ..V.
AAN74572.1 : QQ..V.
ALM00059.1 : QQ..V.
AHSV-2
ref : NEFDFE
AIX10306.1 : R...I.
AIX10307.1 : R...I.
ALM00129.1 : R...I.
ALM00121.1 : R...I.
ALM00123.1 : R...I.
ALM00124.1 : R...I.
ALM00125.1 : R...I.
ALM00126.1 : R...I.
ALM00127.1 : R...I.
UZQ19626.1 : R...I.
AHSV-9
Fig S1. Analysis of the conservation of the 670NEFDFE675 epitope in nine serotypes of AHSV. The dots indicate residues identical to the reference sequence (ref)
